# Investigation of the Nonradiative Photoprocesses of Unnatural DNA Base: 7-(2-Thienyl)-imidazo[4,5-*b*]pyridine (Ds)—A Computational Study

**DOI:** 10.1021/acs.jpca.4c04070

**Published:** 2024-09-16

**Authors:** Paulami Ghosh

**Affiliations:** Department of Chemistry, Georgia State University, Atlanta, Georgia 30303, United States

## Abstract

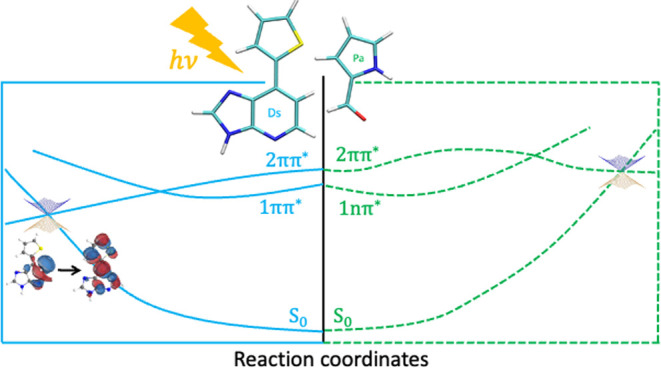

7-(2-Thienyl)-imidazo[4,5-*b*]pyridine (Ds) is an unnatural nucleic acid that forms a stable pair with pyrrole-2-carbaldehyde (Pa) in DNA. This Ds–Pa pair gets stabilized via van der Waals interaction and shape fitting. In our previous study [GhoshP.J. Phys. Chem. A2021, 125, 5556–5561], we investigated the nonradiative photoprocesses of the unnatural DNA base Pa, and also there are some studies on its stability and reactivity in the ground state. But, to consider it as a good unnatural base pair, one has to understand its stability not only in the ground state but also in the excited states after absorbing ultraviolet (UV) radiation. Therefore, in this study, the excited-state photoprocesses of Ds on UV irradiation and its nonradiative decay channels have been investigated using state-of-the-art multireference methods, and this investigation finally leads the molecule to access the minimum energy crossing point (MECP) via a downhill pathway.

## Introduction

To understand the carcinogenic effects upon absorption of ultraviolet (UV) radiation on DNA, it is very important to study the UV-induced photophysics and photochemistry of DNA nucleic acid bases (NABs). NABs are important constituents of life. The hydrogen bonding interaction between NABs such as adenine–thymine (A–T) and guanine–cytosine (G–C) in a Watson–Crick pair is the core of its stabilization along with the DNA double helix.^1−3^ Over the last few decades, there has been significant effort toward expanding the genetic alphabet. The genetic information, or genetic code, is stored within the specific sequence of the DNA helix, which naturally contains only four NABs: adenine (A), thymine (T), cytosine (C), and guanine (G). When RNA is considered, uracil (U) replaces thymine as one of the bases, resulting in a five-base system across both DNA and RNA.^4−6^ Expanding the genetic alphabet beyond these natural bases could potentially increase the capacity for storing genetic information manyfold, which eventually leads to the development of unnatural base pairs (UBPs). This expansion has the potential to revolutionize various fields, such as synthetic biology, biotechnology, and genetic engineering. Consequently, significant research has been directed toward understanding the different interactions that stabilize DNA bases and synthesizing new compounds that can be incorporated into DNA and remain stable in those environments.^7,8^ Examples of successful UBPs include those developed by the Benner and Romesberg groups,^9,10^ which introduced new base pairs such as isoG-isoC and d5SICS-dNaM, respectively.^11,12^

The creation and integration of UBPs into DNA sequences present several challenges such as chemical stability, replication fidelity, compatibility with cellular machinery etc.^13^ Despite these challenges, the expansion of the genetic alphabet holds great promise. It could enable the creation of proteins with novel amino acids, leading to new functionalities and properties that are not possible with the 20 standard amino acids encoded by the natural genetic code.^14^ However, it has also been shown that significantly different chemical entities can be stabilized either by strong hydrogen bonds or by π–π and van der Waals interactions.^7,15,16^

Some UBPs rely on different stabilizing forces such as pyrrole-2-carbaldehyde (Pa) and 7-(2-thienyl)imidazo[4,5-*b*]pyridine (Ds) forming a pair of unnatural nucleic acid bases that are stabilized by van der Waals interactions, hydrophobic effects, and shape complementarity.^17−19^ The hydrophobic nature of the Ds–Pa pair helps them to pair selectively with each other and reduces mispairing, such as forming Ds–Ds pairs.^20,21^ These properties make Ds–Pa a promising candidate for the expansion of the genetic alphabet. Their unique stabilizing interactions offer a different mechanism compared to the natural hydrogen-bonded base pairs, potentially increasing the versatility and functionality of synthetic genetic systems.

Two important aspects that need to be studied to understand the stability and instability of UBPs are their photoactivity and photochemical stability. People have extensively studied the photoprocesses and nonradiative deactivation mechanism of DNA bases experimentally as well as computationally.^22−27^ The excited photoprocesses and nonradiative pathways present in NABs have been widely studied by many research groups.^28−33^ Not only NABs but also UBPs have been extensively studied experimentally as well as theoretically. The effect of solvation on the excitation of two such UNBs called d5SICS and dNaM has been studied by Pollum et al.^34^ A series of experimental works have been done to understand the photochemical properties of d5SICS, dNaM, and dTPT3, and it has been found that the triplet state plays a crucial role in causing photodamage in UBP-containing DNA.^35,36^ These experimental findings about d5SICS and dNaM have been theoretically supported by Bhattacharyya and Datta,^37^ who used time-dependent density functional theory (TD-DFT) and the multistate complete active space self-consistent field followed by second-order perturbative correction (SA-CASSCF/MS-CASPT2) level of theories. QM/MM studies on dTPT3 have been done by Cui et al.,^38^ who investigated the two main excited-state relaxation pathways that populate the triplet state. They also investigated the photophysical processes of the unnatural base Z using the highly accurate multistate complete active space second-order perturbation (MS-CASPT2) theory.^39^

Conical intersections (CIs) are essential for understanding the photostability and nonradiative decay processes of molecules, particularly in relation to DNA bases and their photochemical behavior. CIs can be categorized into two main types based on their topographical parameters:^40^ sloped CIs, which are associated with photostable molecules, and peaked CIs, which are linked to a higher likelihood of photoproduct formation. Molecules exhibiting sloped CIs tend to be photostable, as they are more likely to regenerate their ground state after reaching the CI region.^41−46^ This characteristic is commonly observed in most DNA bases, contributing to their overall photostability. In contrast, molecules with peaked CIs are more prone to forming photoproducts, which can lead to significant chemical changes or damage.^47,48^ The presence of sloped CIs in DNA bases is a key factor in their ability to efficiently dissipate absorbed UV energy through nonradiative decay back to the ground state, thereby protecting DNA from UV-induced damage.^30,49^ The study of CIs holds important implications for various fields, including the design of photostable molecules for applications in photobiology and materials science as well as for predicting the photochemical behavior of new compounds, including potential unnatural base pairs in expanded genetic systems.

**Figure 1:**
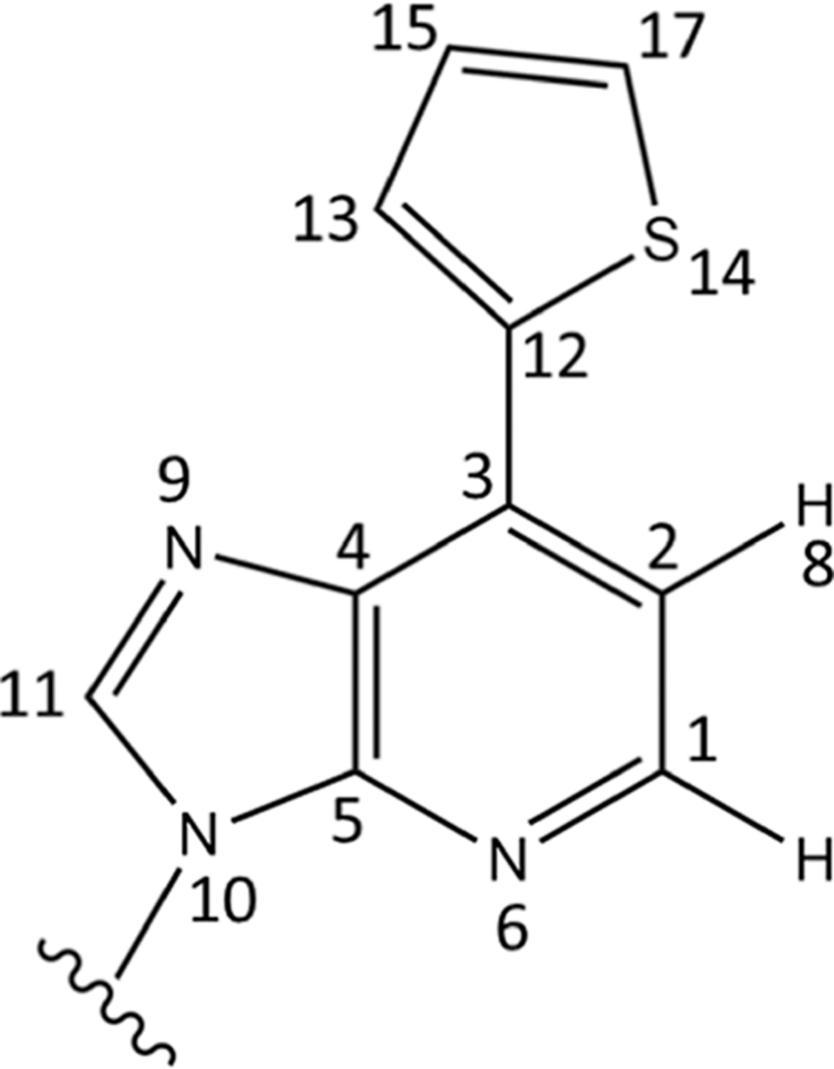
Chemical structure of Ds.

In this work, the photoprocesses of the unnatural base Ds of the Ds-Pa pair of DNA strands have been investigated. The entire photo-deactivation pathway of the Ds molecule involving low-lying singlet excited states has been elucidated, followed by calculations of the topographical parameters around S_0_/S_1_ and the minimum energy CI to understand the photostability of Ds (Figure 1). It will be shown using state-of-the-art multireference methods that the significant distortion within the ring is the motion that will finally lead to an accessible conduit for the molecule to relax. The topographical parameters of the minimum energy CI have been defined in the Computational Details section of the manuscript.

## Computational Details

**Figure 2:**
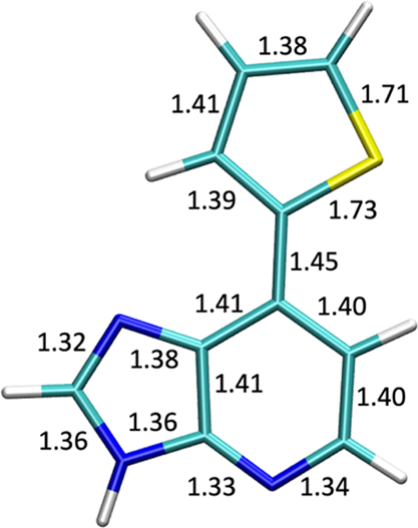
7-(2-Thienyl)-imidazo[4,5-*b*]pyridine at the Franck–Condon (FC) region optimized at the RI-MP2/cc-pVTZ level of theory.

The ground-state (S_0_) geometry of 7-(2-thienyl)-imidazo[4,5-*b*]pyridine (Ds) given in Figure 2 is optimized with resolution-identity Møller–Plesset perturbation (RI-MP2) theory using the cc-PVTZ basis set. This has also been optimized with SA-CASSCF/6-31+g(d) level of theory for the comparison. The Cartesian coordinates of the S_0_ minima of Ds at two different levels of theories are given in the Supporting Information (SI) [Section S1].

The vertical excitation energies (VEEs) of Ds have been calculated at different levels of theories such as (i) equation of motion coupled cluster singles and doubles (EOM-EE-CCSD),^50^ (ii) state-averaged complete active space self-consistent field theory (SA-CASSCF),^51^ and (iii) multistate complete active space self-consistent including second-order perturbation theory (MS-CASPT2).^52^ It has always been noticed that for natural NABs, excited states having ππ*, *n*π*, and, in some cases, πσ* transitions are important to explain the photophysics.^37,53^ Keeping these in mind, a (10e,12o) active space consisting of 1 nonbonding orbital, 4π-orbitals, 1σ* orbitals, and 6π* orbitals (shown in Section S3 of SI) has been chosen for all of the single-point calculations. 6-roots MS-CASPT2 has been performed to incorporate dynamic correlation by using 6-roots SA-CASSCF wave functions as the reference. To avoid the intruder state problem in calculating the perturbative energy, a level shift of 0.3 au has been used. No IPEA shift has been used in these calculations. S_0_ has been reoptimized with (10e,12o) 3-roots SA-CASSCF/6-31+g(d) level of theory for comparison, which gives a similar geometry to that obtained from RI-MP2/cc-pVTZ level of theory. The first optically active ππ* state has been optimized with the 3-roots SA-CASSCF/6-31+g(d) level of theory (Cartesian coordinate is given in Section S2 of SI). Three roots have been used for optimization at SA-CASSCF level of theory and 6 roots have been used for all single-point calculations at SA-CASSCF and MS-CASPT2 levels of theories.

A minimum energy crossing point (MECP)^54^ has been searched between S_0_ and S_1_ with the 2-roots SA-CASSCF method, where the (8e,6o) active space at 6-31+g(d) basis set has been used. This has been done by calculating the gradient^55^ of both the excited states using the couple-perturbed multiconfigurational self-consistent field (CP-MCSCF)^56^ theory in Molpro.^57^ The orbitals included in the (8e,6o) active space are shown in Section S3 of the SI. 3π, 1 nonbonding, and 2π* orbitals have been chosen for MECP optimization to include both ππ* and *n*π* states. The Cartesian coordinate of S_0_/S_1_ MECP is given in Section S4 of SI. Intermediate geometries between the Franck–Condon (FC) region and MECP have been generated using the linear interpolation of Cartesian coordinates and will be denoted as linearly interpolated internal coordinates (LIIC). LIIC potential energy surfaces (LIIC-PES) have been constructed with (10e,12o) SA-CASSCF/6-31+g(d) level of theory followed by MS-CASPT2 level of theory. The minimum energy pathway (MEP) of Ds was constructed by constraint optimizations at different C_12_–C_2_–C_3_–H_8_ dihedral angles. The dihedral angle constrained optimization has been performed with the time-dependent density functional theory (TD-DFT) method using CAM-B3LYP^58^ exchange functional at 6-311++g(d,p) basis set. The MEP has been subsequently constructed with single-point calculations at TD-DFT optimized geometries with SA-CASSCF/6-31+g(d) followed by MS-CASPT2/6-31+g(d) to include dynamic correlation.

Two-dimensional (2D) surfaces have been created around the MECP from the four topographical parameters to understand the fate of the molecule on reaching the MECP.^59^ The linearly approximated adiabatic energies of S_0_ and S_1_ states around the MECP have been created from the following equation
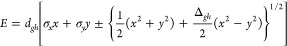
1where *d_gh_*, Δ*_gh_*, σ_*x*_, and σ_*y*_ are the four topographical parameters as described in ref (40). *d_gh_* denotes the pitch of the cone, Δ*_gh_* is the deviation from cylindrical symmetry, and σ_*x*_ and σ_*y*_ are the tilts of the cones away from the vertical direction. Here *g⃗* signifies the energy gradient difference vector and *h⃗* is the nonadiabatic coupling vector. These can be defined as follows
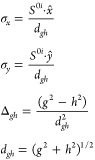
where  and , *S*^0*i*^ is the gradient sum vector, where *i* represents the state at which the ground state has an MECP. *x̂* and *ŷ* are the unit vectors based on Schmidt-orthogonalized energy gradient difference vector (*g*^0*i*^) and nonadiabatic coupling vector (*h*^0*i*^).

The EOM-EE-CCSD and TD-DFT calculations have been performed with the quantum chemistry software package Q-Chem^60^ and the active space-based calculations have been performed with Molpro.^57^

## Results and Discussion

The ground-state optimized geometry of the Ds molecule is almost planar in structure [Figure 2]. The molecule is kept in C_1_ symmetry throughout.

### Vertical Excitation Energies (VEEs)

**Figure 3:**
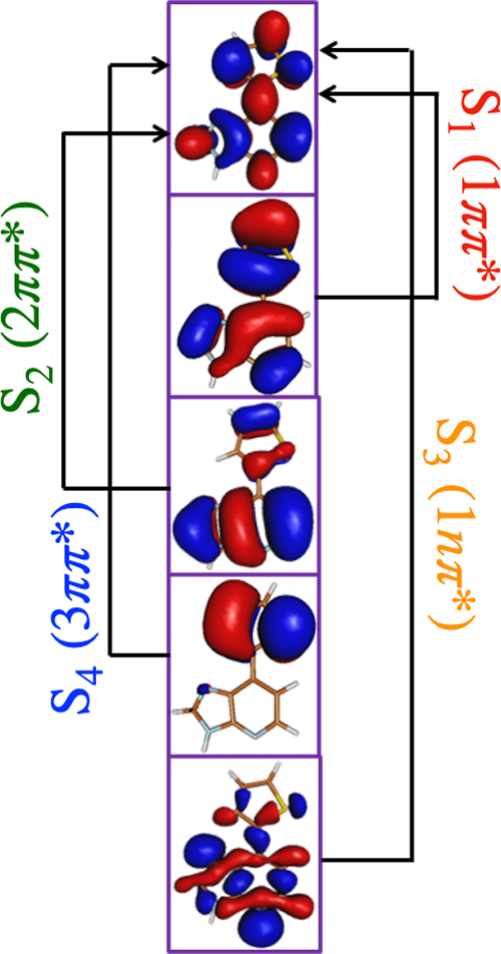
Orbitals involved in the lowest four excited states of Ds calculated at the EOM-EE-CCSD level of theory.

Table 1 shows the calculated VEEs of Ds at S_0_ minima optimized at the RI-MP2/cc-pVTZ level of theory. VEEs calculated at EOM-EE-CCSD and (10e,12o) 6-roots SA-CASSCF followed by 6-roots MS-CASPT2 levels of theories show a similar trend of low-lying excited states of Ds at S_0_ minima. The first singlet excited state (S_1_) and fourth singlet excited state (S_4_) are the optically active ππ* states having a higher oscillator strength (O.S.). The second singlet excited state (S_2_) is another ππ* state having a lower O.S. and the third singlet excited state (S_3_) is dark *n*π* in nature. The optically active ππ* S_1_ excitation energy calculated by the (10e,12o) 6-roots MS-CASPT2 method is 4.79 eV (∼259 nm) and that with the EOM-EE-CCSD method is 4.56 eV (∼272 nm). These are in close agreement with the range of experimental λ_max_ of DNA NABs (250 to 280 nm). The orbitals involved in the low-lying excitations of Ds calculated at the EOM-EE-CCSD/6-31+g(d) level of theory are shown in Figure 3.

**Table 1:** VEEs (in eV) of Ds with (10e,12o) 6-Roots SA-CASSCF and MS-CASPT2/6-31+g(d) Levels of Theories, Which Are Compared with EOM-EE-CCSD/6-31+g(d) Results^a^

state	SA-CASSCF (10e,12o)	MS-CASPT2 (10e,12o)	EOM-EE-CCSD	character
S_1_	**5.45**	**4.79**^b^	**4.56**	1ππ*
	(0.3218)	(0.3054)	(0.5184)	
S_2_	5.71	5.22	4.66	2ππ*
	(0.0876)	(0.0798)	(0.066)	
S_3_	5.78	5.24	5.23	1*n*π*
	(0.0094)	(0.0063)	(0.0011)	
S_4_	**6.23**	**5.47**	**5.32**	3ππ*
	(0.6802)	(0.6520)	(0.1197)	

a The oscillator strength (O.S.) of the excited states are given in parentheses. The optically active ππ* states for every level of theory are given in bold font.

b The calculated absorption spectra of Ds are compared with the λ_max_ of native DNA at 20/∼g/mL in 0.15 M NaC1 (pH 7.0); see ref (61).

### Excited-State Surface and MECPs

**Figure 4:**
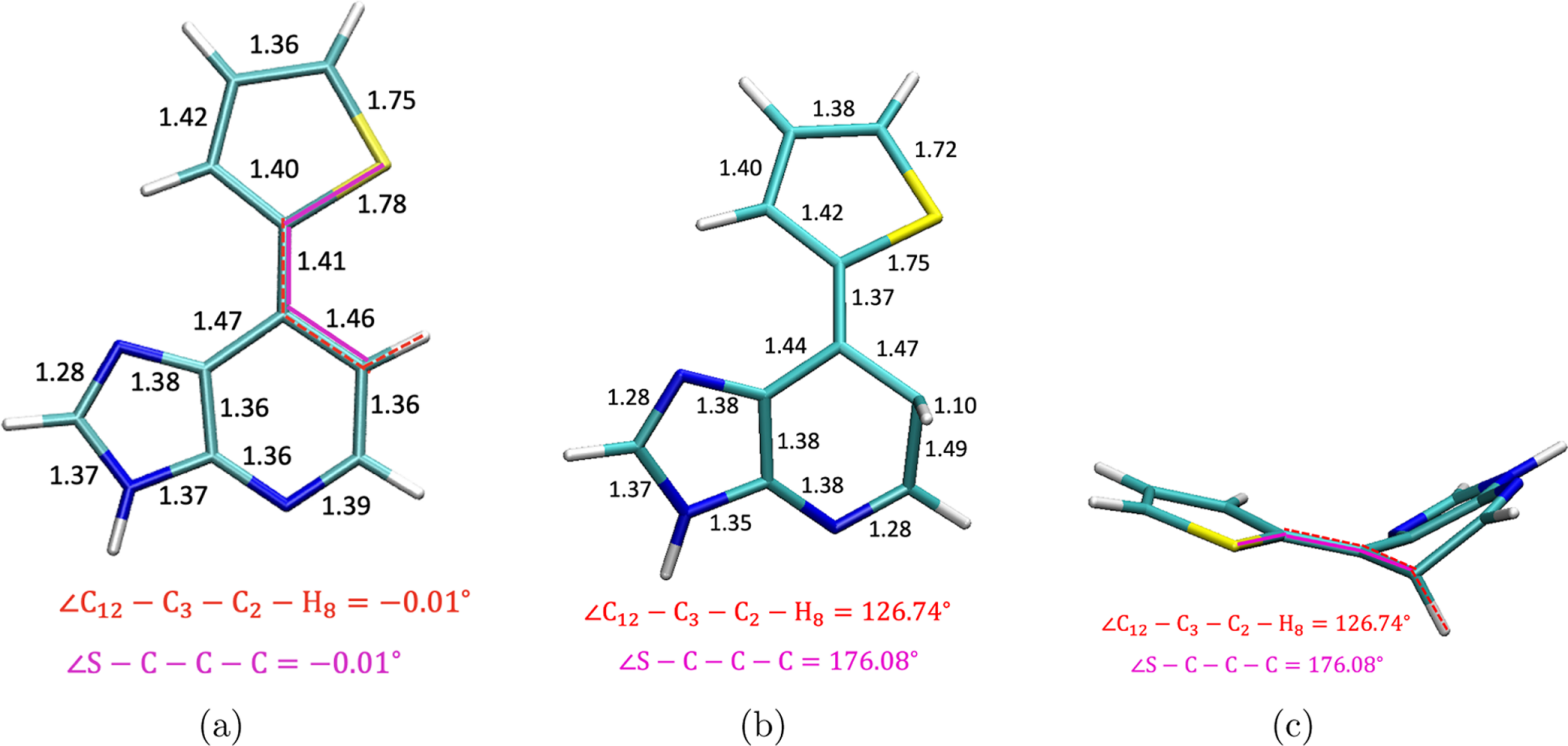
(a) Ds at S_1_ minima. (b) S_0_–S_1_ MECP of Ds. (c) Out-of-plane view of the S_0_–S_1_ MECP of Ds.

Upon UV absorption, Ds molecules will populate the optically active S_1_ state having Ms-CASPT2 excitation energy 4.79 eV followed by S_1_ minima, which is very near to the FC region given in Figure 4.

**Figure 5:**
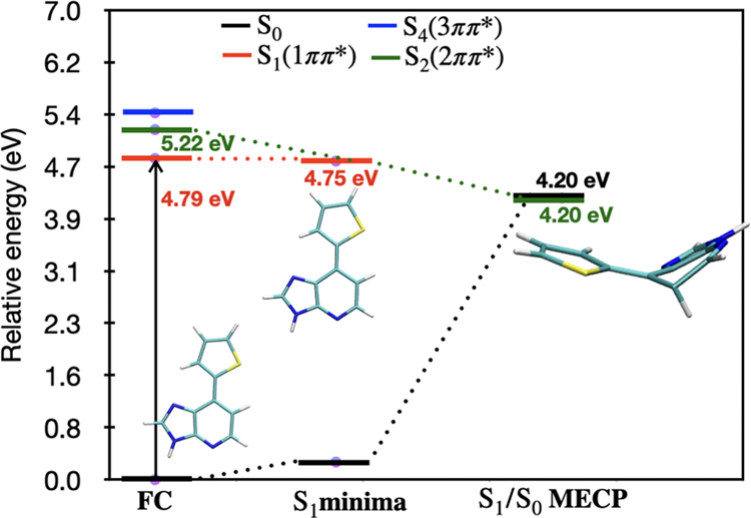
Schematic diagram of Ds connecting the FC region to the MECP via S_1_ minima. All of the relative energies shown in this figure have been calculated with (10e,12o) MS-CASPT2 level of theory on the respective optimized geometries.

To understand the nonradiative processes present in Ds, MECP optimization between S_1_ and S_0_ has been computed. One MECP between S_1_ and S_0_ is located for Ds, which is nonplanar in nature with an out-of-plane C–H bond of a six-member ring. The dihedral angle between C_12_–C_3_–C_2_–H_8_ has been changed from −0.01° at S_1_ minima to 126.74° at MECP. On analyzing the nature of orbitals involved in MECP, it is noticed that S_1_ at MECP is no longer pure ππ* in nature but with a mix of *n*π* character, which is expected from the nonplanarity introduced in the structure of MECP. The MECP is lower in energy than the FC region by ∼0.6 eV (∼14 kcal/mol). A schematic diagram connecting FC to MECP via the S_1_ minima of Ds is shown in Figure 5.

### Excited-State Deactivation Mechanism

**Figure 6:**
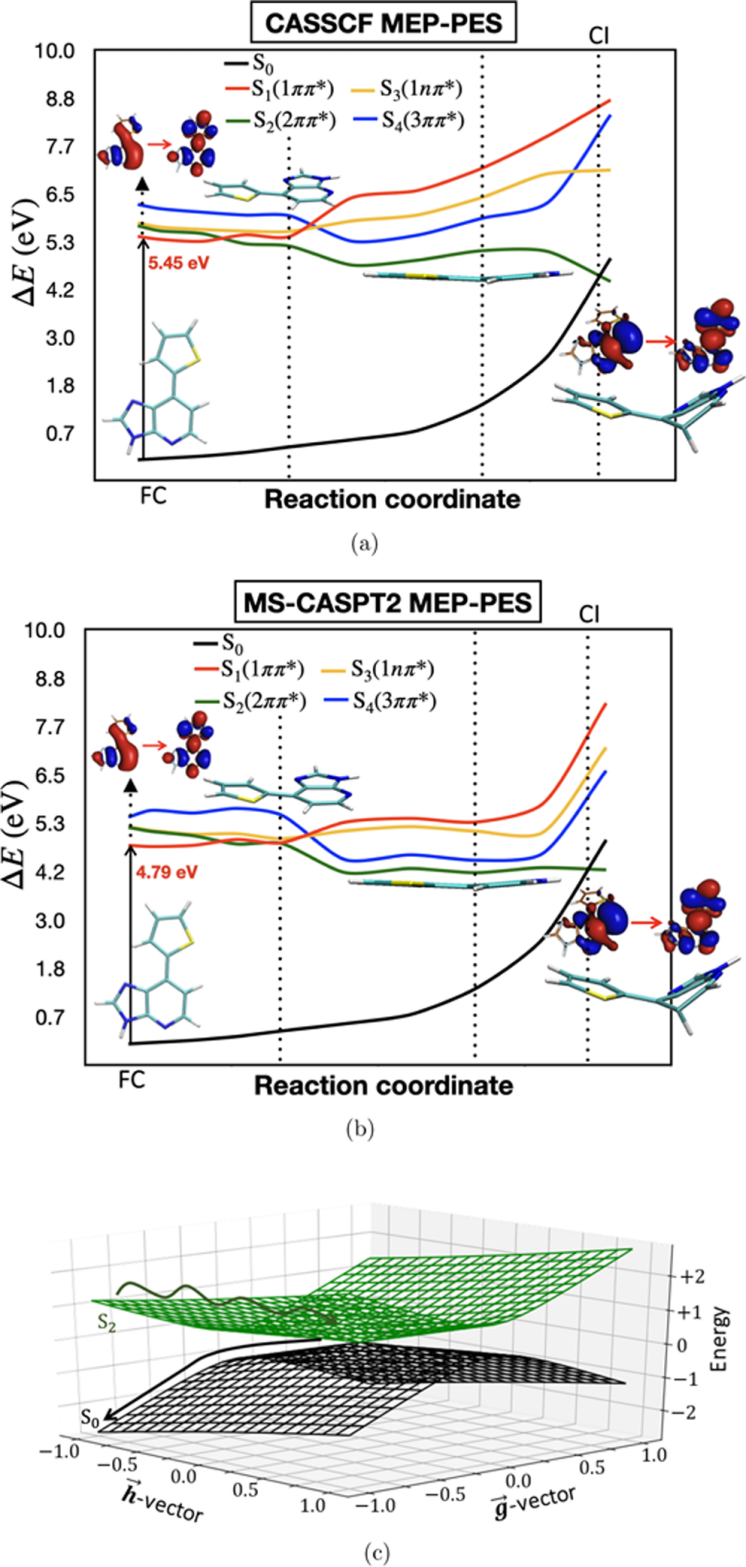
(a) 6-roots SA-CASSCF MEP-PES of Ds along the reaction coordinate. (b) 6-roots MS-CASPT2MEP of Ds between FC to MECP along the reaction coordinate C_12_–C_3_–C_2_–H_8_ dihedral angle: S_1_ (1ππ*) (red), S_2_ (2ππ*) (green), S_3_ (1*n*π*) (yellow), and S_4_ (3ππ*) (blue). The orbitals shown at PESs are the orbitals involved in the π–π* state at the corresponding geometries. (c) 2D surfaces around MECP.

From the schematic diagram [Figure 5], it is clear that the S_0_/S_1_ MECP is ∼0.6 eV lower in energy than FC. But this does not necessarily imply that the deactivation pathway of Ds will be barrier-less. Therefore, the question that comes to mind is whether there is any barrier along the pathway from FC to MECP or not. To answer this question, LIIC-PES has been created connecting FC to MECP via S_1_ minima. The LIIC-PES has a very high barrier, which is expected as the MECP is highly nonplanar and there is lot of mixing in the orbital characters involved in the lower-lying excited states. The MS-CASPT2 LIIC-PES is given in Section S5 of SI. To understand this phenomenon in detail, the MEP for Ds along the C_12_–C_3_–C_2_–H_8_ dihedral angle given in Figure 6(b) has been constructed. From Figure 6(b), it is noticed that after photoexcitation to S_1_ state having excitation energy 4.79 eV, there is a crossing between S_1_ (1ππ*) and S_2_ (2ππ*) states by which 2ππ* state gets populated. If we look at the π orbital involved in 2ππ* state and follow the energetics of the involved π and π* orbitals starting from FC to MECP, it can be noticed that due to nonplanarity in the purine-type ring, as the conjugation breaks, the π orbital gets highly destabilized with a shifting of the electron density to a six-member ring and the π* orbital gets stabilized. As a result of that, the energy of the 2ππ* state decreases, followed by getting involved in the MECP with the S_0_ state. The (10e,12o) 6-roots SA-CASSCF energetics of π and π* orbitals involved in 2ππ* state is given in Section S9 of SI. But here, the 2ππ* state is no longer a pure ππ* state, it has a mixed *n*π*/ππ* character. It is also important to notice from Figure 6(b) that the pathway along the C_12_–C_3_–C_2_–H_8_ dihedral angle to reach MECP from FC is barrier-less with a flat surface.

The 2D surfaces around the S_0_/S_1_ MECP of Ds molecule are shown in Figure 6(c). The values of topographical parameters such as σ_*x*_ and σ_*y*_ (tilts of the cones away from the vertical direction) and Δ_*gh*_ (deviation from cylindrical symmetry) are +ve (given in Section S7 of SI), which means that the MECP or the CI is a sloped one.^46^ The surface of the sloped CI is tilted toward the negative direction *h⃗*-vector and it is symmetrical about the +*g⃗*-vector. Now, the +*h⃗*-vector corresponds to a slow in-plane motion, where +*g⃗*-vector corresponds to the out-of-plane motion shown in S6 of SI. As the surface is tilted toward −*h⃗*-vector and symmetrical about the *g⃗*-vector, it implies that the molecule will have a higher probability of going back to the S_0_ minima, which makes the unnatural base Ds a photostable one. This will be discussed in details in the Conclusions.

## Conclusions

The unnatural base Ds is planar in nature, and it has several optically active low-lying π–π* states and one dark *n*–π* state. An active space consisting of 10 electrons in 12 orbitals has been chosen depending on the orbitals involved in the low-lying excited states according to the EOM-EE-CCSD calculation at S_0_ minima. The (10e,12o) 6-roots MS-CASPT2 excitation energy of S_1_ is 4.79 eV, which is in good agreement with the experimental absorption spectra of natural DNA bases. It is also noticed that the S_1_ minima of the Ds molecule is very close to FC geometry.

One S_1_/S_0_ MECP is obtained for Ds, which is nonplanar with a ring-puckered structure [Figure 4(b,c)]. This is comparable with the MECP observed in the case of the natural DNA bases adenine.^28,29^ The MECP of Ds is ∼0.6 eV (∼14 kcal/mol) lower in energy than the FC region. The MEP-PES of Ds was constructed to estimate the energy barrier required for accessing S_1_/S_0_ MECP. A barrier-less flat PES is obtained.

The *g⃗* and *h⃗*-vectors of Ds around S_1_/S_0_ MECP has been given in SI (Section S6). The 2D surface around the MECP of Ds is tilted toward the negative direction of the *h⃗*-vector and symmetric about the *g⃗*-vector. Analysis of the normal modes along both the *g⃗* and *h⃗*-vectors and the sloped CI indicated that after reaching MECP, the molecule will have a higher probability of being in the photoprotection mode, i.e., regeneration of S_0_ will occur, which makes the Ds molecule a photostable unnatural base . There is a study by Malhado et al.^62^ that showed that tilt angles (σ_*x*_ and σ_*y*_) of the 2D surfaces do not tell us about the transition probability of molecules from S_1_ to S_0_, and as a result of that, one cannot infer that peaked conical intersections will have a higher transition probability than sloped conical intersections. This can be explained by the velocity of the molecules before reaching the CI region, which depends on the kinetic energy of the molecules. When the FC region of the excited state is much higher in energy than the CI region, then the molecules will reach at the CI with a higher kinetic energy than the pathway having a lower energy difference between the FC and CI regions. But if the pathway contains an activation barrier to reach the CI, then that extra kinetic energy will be used to cross the barrier, whereas the pathway with molecules having a lower kinetic energy and no activation barrier will be more efficient for the molecules to reach the CI region. In case of Ds molecules, the latter justification has been noticed, i.e., in this case, deactivation surface being flat and barrier-less, there will be a higher probability for the molecules to reach the MECP and if they reach the MECP, from the analysis of *g* and *h*-vectors (in the last paragraph of Results and Discussion section), one can conclude that there will be a higher probability of photoprotection, which makes Ds a photostable unnatural base.

We can compare the nonradiative pathway of the Ds molecule with that of purine bases like adenine, guanine etc. Adenine shows a similar type of MECP where one C–H bond of the six-member ring gets out-of-plane,^28^ as obtained for Ds [Figure 4(b,c)]. From the literature studies,^30−33^ it is observed that purine bases exhibit energetically favorable and barrier-less pathways, which have a faster deactivation time. In the case of Ds, a similar MECP along with a barrier-less deactivation pathway is obtained, where the nonradiative decay pathway is mainly characterized by a mixing of ππ*/*n*π* states.^63^

In our previous study,^64^ we investigated the nonradiative photoprocesses of pyrrole-2-carbaldehyde (Pa), which forms a stable pair with Ds in DNA and acts as a Ds–Pa unnatural base pair. We showed that the deactivation pathway of Pa is very similar to the deactivation pathways of pyrimidine bases (thymine, uracil, cytosine), which are mainly characterized by richer PESs. In this study, it is noticed that Ds shows a similar barrier-less deactivation pathway as purine bases. From these two individual studies of nonradiative decay, one can expect that though Pa has a small energy barrier (∼4 kcal/mol), the Ds–Pa pair will have an energetically accessible deactivation pathway as Ds has a barrier-less deactivation pathway. But in both cases, flat surfaces of the involved excited states in MECP are noticed, from which one can expect a comparatively longer deactivation time of the Ds–Pa pair.

## Supporting Information Available

Cartesian coordinates of S_0_ minima of Ds at various levels of theories; cartesian coordinates of S_1_ minima of Ds molecule optimized at (10e,12o) 3SA-CASSCF/6-31+g(d) level of theory; CASSCF orbitals used in (8e,6o)/6-31+g(d) active space to search MECP and (10e,12o)/6-31+g(d) active space to calculate VEEs of Ds molecule; cartesian coordinates of optimized geometries of S_1_–S_0_ MECP of Ds; MS-CASPT2 LIIC-PES along two MECP of Ds molecule; g⃗ and *h⃗*-vectors and the values of topographical parameters around MECP of Ds molecule; OPDM of S_0_ and S_1_ at S_0_/S_1_ MECP of Ds; (10e,12o) 6-roots SA-CASSCF energy (in Hartree) of involved π and π* orbitals in 2*ππ** state along the MEP-PES, and effect of basis sets on VEEs calculated at TD-DFT/b3lyp level of theory at S_0_ minima (PDF)
