# Supplementary material for: Investigation of the Nonradiative Photoprocesses of Unnatural DNA Base: 7-(2-Thienyl)-imidazo[4,5-b]pyridine (Ds)—A Computational Study
Source: J Phys Chem A. 2024 Sep 16;128(38):8065–71. doi: 10.1021/acs.jpca.4c04070 (PMC11440586; doi:10.1021/acs.jpca.4c04070)
Supplement: Supplementary file 1 — jp4c04070_si_001.pdf [file jp4c04070_si_001.pdf]

Supporting Information For Publication: Investigation of the non-radiative photo-processes of unnatural DNA base: 7-(2-thienyl)-imidazo[4,5-b]pyridine (Ds) - A computational study

Paulami Ghosh*, †

Email: paulamighosh1991@gmail.com
†Department of Chemistry, Georgia State University, Atlanta 30303, Georgia, USA.
September 7, 2024

S1 Cartesian coordinate of S_0 minima

S1.1 RI-MP2/cc-pVTZ level of theory

C	1.66368947	-2.30607020	-0.00132302
C	0.35079585	-1.81616336	-0.00454632
C	0.06744827	-0.44634917	-0.00451025
C	1.20852078	0.38050891	-0.00076509
C	2.47616667	-0.24340606	0.00226453
N	2.76456154	-1.54191407	0.00213478
H	1.83052971	-3.37559252	-0.00154136
H	-0.45913889	-2.53352288	-0.00698482
N	1.34405059	1.75275063	0.00047294
N	3.37682470	0.79596231	0.00549818
C	2.65076030	1.94870299	0.00424288
C	-1.28292074	0.08246286	-0.00787352
C	-1.68793887	1.41122914	-0.00549437
S	-2.65660962	-0.96692296	-0.01437894
C	-3.08809041	1.55648912	-0.01013478
H	-0.97140785	2.21759972	-0.00097324
C	-3.75037400	0.34710318	-0.01538148
H	-3.60067139	2.50659941	-0.00943269
H	-4.81217064	0.16494855	-0.02005598

H	4.37881517	0.70283455	0.00804960
H	3.12347121	2.91719328	0.00579276

S1.2 (10e,12o) 3-roots SA-CASSCF/6-31+g(d) level of theory

C	1.6723769824	-2.3048816194	0.0017966041
C	0.3560212644	-1.8176712564	0.0020306275
C	0.0877457019	-0.4478423212	0.0017673663
C	1.2297391198	0.3721240217	0.0011606068
C	2.4745590416	-0.2334091631	0.0008218657
N	2.7542733368	-1.5357430186	0.0010558867
H	1.8440805466	-3.3658638672	0.0018578569
H	-0.4455631133	-2.5319767185	0.0026721558
N	1.3816522100	1.7499208475	0.0007481581
N	3.3781984747	0.7970019684	0.0001462914
C	2.6509516240	1.9438821673	0.0001736889
C	-1.2850413383	0.0818601588	0.0019888459
C	-1.6807691147	1.3908378309	0.0020960704
S	-2.6816134611	-0.9865045593	0.0022965974
C	-3.1106094865	1.5513796459	0.0022096808
H	-0.9876799083	2.2063183698	0.0016088580
C	-3.7716192959	0.3637721918	0.0030385491
H	-3.6007881329	2.5063503279	0.0021693272
H	-4.8301076764	0.2002210678	0.0017386234
H	4.3689511890	0.7084130482	-0.0001158760
H	3.1216033321	2.9061985587	-0.0002617843

S2 Cartesian coordinate of S_1 minima optimized at (10e,12o) 3-roots SA-CASSCF/6-31+g(d) level of theory

C	1.5974842719	-2.4008168971	0.0017630391
C	0.3418978302	-1.8739452392	0.0019697100
C	0.0554404064	-0.4438306322	0.0016527212
C	1.2617739105	0.3968932958	0.0010730140
C	2.5017195324	-0.1643662460	0.0008065019
N	2.6642093257	-1.5127395934	0.0010454312
H	1.8277426908	-3.4445084092	0.0019936845
H	-0.4702525233	-2.5750641419	0.0025312702
N	1.4130144971	1.7675186482	0.0006984090
N	3.4091743077	0.8367927637	0.0002586102
C	2.6723595767	1.9961935851	0.0002377423
C	-1.2591693392	0.0603622436	0.0018892207

C	-1.6794231461	1.3946006948	0.0017941618
S	-2.6814815483	-1.0066359190	0.0024507046
C	-3.0962501454	1.5440934237	0.0021442016
H	-0.9887505841	2.2112671782	0.0014228778
C	-3.7681782130	0.3588219921	0.0027298023
H	-3.5883168307	2.4985763681	0.0020686213
H	-4.8270819083	0.2024479077	0.0025634846
H	4.3987862440	0.7517275542	0.0000494750
H	3.1416629408	2.9569991039	-0.0001426831

S3 CASSCF natural orbitals used in different calculations

S3.1 (10e,12o)/6-31+g(d) active space to calculate VEEs

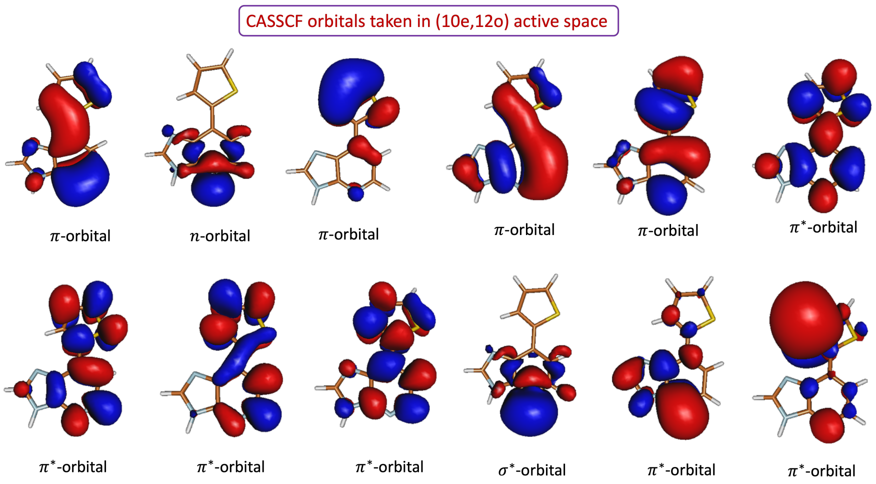

Figure S1: CASSCF orbitals taken in (10e,12o) active space to calculate VEEs and for constructing LIIC-PES and MEP PES of Ds.

S3.2 (8e,6o)/6-31+g(d) active space to find S_0/S_1 MECP

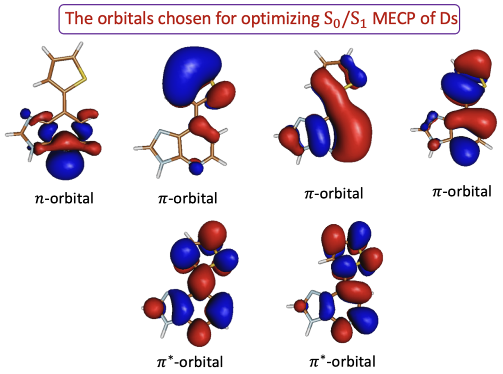

Figure S2: CASSCF orbitals at S_0/S_1 MECP of Ds.

S4 Cartesian coordinate of S_0/S_1 MECP of Ds

C	1.3694269098	-2.2749125677	0.2273894244
C	0.3271338131	-1.7865745513	-0.7115156424
C	0.0032664680	-0.3622262435	-0.5351881575
C	1.2167226393	0.4128631423	-0.4903415137
C	2.2371791633	-0.2273809927	0.1757535403
N	2.3147073326	-1.5224546400	0.6541452422
H	1.4327482593	-3.3194859710	0.4837034350
H	0.8375596526	-1.8788816589	-1.6793744593
N	1.5289203125	1.7442339249	-0.6663868289
N	3.1794391433	0.7190049166	0.3819458130
C	2.7006980687	1.8813118856	-0.1584097484
C	-1.2631530680	0.0847708121	-0.2524119967
C	-1.6854190985	1.4028620473	0.0466942825
S	-2.6140310752	-1.0314361962	-0.1824679401
C	-3.0449274327	1.4686517504	0.3887127997
H	-1.0109150126	2.2351718584	0.0298534103
C	-3.6718440686	0.2370919698	0.3137061638
H	-3.5505485795	2.3718209520	0.6721930757
H	-4.7020173013	0.0318171762	0.5245391779
H	4.0490146957	0.5820715980	0.8470389826
H	3.2723510305	2.7861242528	-0.1445192517

S5 MS-CASPT2 LIIC-PES of Ds

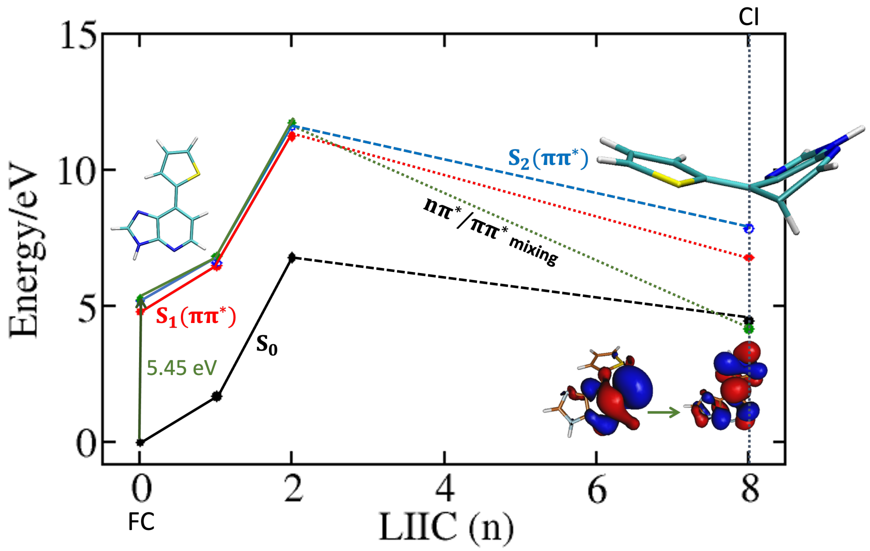

Figure S3: MS-CASPT2 LIIC-PES of Ds starting from FC to MECP

S6 g and h-vectors around MECP

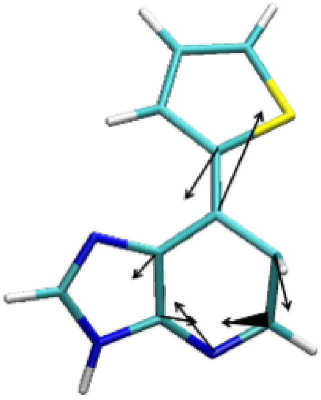

(a) g-vector around MECP

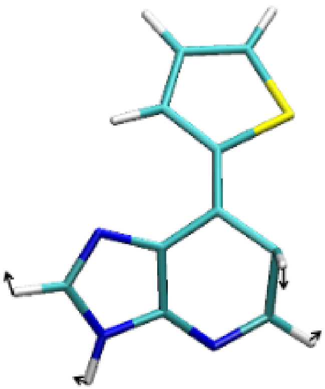

(b) h-vector around MECP

Figure S4: g and h-vectors around S_0/S_1 MECP of Ds

S7 Topographical parameters around MECP of Ds

	value
d_{gh}	0.1984
Δ_{gh}	0.8954
σ_x	0.0400
σ_y	0.3618

S8 OPDM of S_0 and S_1 at S_0/S_1 MECP of Ds

ONE-PARTICLE DENSITY MATRIX FOR STATE 1

	1	2	3	4	5	6	7	8
1	1.9227648	0.0088946	0.0035878	-0.0182227	0.0091971	0.0506596	0.0164113	-0.0102987
2	0.0088946	1.9342958	0.0102661	-0.0178410	-0.0052712	-0.0125319	-0.0821365	-0.0100543
3	0.0035878	0.0102661	1.9715822	0.0180735	-0.0566059	-0.0364171	0.0266681	0.0005096
4	-0.0182227	-0.0178410	0.0180735	1.8690196	0.0791159	0.1223362	-0.1249889	0.0496078
5	0.0091971	-0.0052712	-0.0566059	0.0791159	0.9851897	0.0771422	0.1541566	0.0446408
6	0.0506596	-0.0125319	-0.0364171	0.1223362	0.0771422	0.9803467	0.1327791	-0.0425763
7	0.0164113	-0.0821365	0.0266681	-0.1249889	0.1541566	0.1327791	0.1649095	-0.0090793
8	-0.0102987	-0.0100543	0.0005096	0.0496078	0.0446408	-0.0425763	-0.0090793	0.0744583
9	0.0077934	-0.0219154	-0.0014402	0.0290435	0.0269171	0.0420172	0.0084409	0.0112441
10	-0.0017524	0.0108055	0.0107737	-0.0079269	0.0926359	-0.0189057	0.0138939	-0.0010991
11	-0.0021817	0.0000214	0.0029637	0.0033982	-0.0175706	-0.0028938	-0.0034320	0.0020150
12	0.0009501	0.0011394	0.0005407	0.0006882	-0.0045133	0.0123167	0.0146646	-0.0052018

	9	10	11	12
1	0.0077934	-0.0017524	-0.0021817	0.0009501
2	-0.0219154	0.0108055	0.0000214	0.0011394
3	-0.0014402	0.0107737	0.0029637	0.0005407
4	0.0290435	-0.0079269	0.0033982	0.0006882
5	0.0269171	0.0926359	-0.0175706	-0.0045133
6	0.0420172	-0.0189057	-0.0028938	0.0123167
7	0.0084409	0.0138939	-0.0034320	0.0146646
8	0.0112441	-0.0010991	0.0020150	-0.0052018
9	0.0631741	-0.0015551	0.0011873	-0.0049416
10	-0.0015551	0.0123671	0.0009706	-0.0000720
11	0.0011873	0.0009706	0.0158321	-0.0003425
12	-0.0049416	-0.0000720	-0.0003425	0.0060600

(a) OPDM of S_0

ONE-PARTICLE DENSITY MATRIX FOR STATE 2

	1	2	3	4	5	6	7	8
1	1.9385868	0.0039050	0.0006296	-0.0088695	-0.0071462	-0.0090005	-0.0015766	-0.0060440
2	0.0039050	1.9517073	0.0104895	-0.0083519	0.0022696	-0.0395568	-0.0305258	-0.0146841
3	0.0006296	0.0104895	1.9723936	0.0161758	-0.0036783	0.0341848	0.0090555	0.0163169
4	-0.0088695	-0.0083519	0.0161758	1.8857462	-0.0303716	-0.1374786	-0.0607462	0.0138275
5	-0.0071462	0.0022696	-0.0036783	-0.0303716	1.8855106	-0.1290703	-0.1152466	0.0921350
6	-0.0090005	-0.0395568	0.0341848	-0.1374786	-0.1290703	0.1351717	0.0093988	-0.0099259
7	-0.0015766	-0.0305258	0.0090555	-0.0607462	-0.1152466	0.0093988	0.0883124	-0.0006935
8	-0.0060440	-0.0146841	0.0163169	0.0138275	0.0921350	-0.0099259	-0.0006935	0.0522532
9	-0.0150450	-0.0057950	0.0009987	0.0152988	-0.0327999	-0.0079448	0.0018625	0.0007496
10	-0.0018208	-0.0030862	-0.0080675	0.0027989	-0.1497672	0.0126897	0.0228661	-0.0122096
11	-0.0014079	0.0032283	0.0268799	0.0019446	0.0187247	0.0010119	-0.0021606	0.0022048
12	0.0017855	-0.0136195	0.0024589	-0.0126177	-0.0072114	0.0034926	0.0104449	0.0001246

	9	10	11	12
1	-0.0150450	-0.0018208	-0.0014079	0.0017855
2	-0.0057950	-0.0030862	0.0032283	-0.0136195
3	0.0009987	-0.0080675	0.0268799	0.0024589
4	0.0152988	0.0027989	0.0019446	-0.0126177
5	-0.0327999	-0.1497672	0.0187247	-0.0072114
6	-0.0079448	0.0126897	0.0010119	0.0034926
7	0.0018625	0.0228661	-0.0021606	0.0104449
8	0.0007496	-0.0122096	0.0022048	0.0001246
9	0.0319386	0.0039940	0.0008035	-0.0035791
10	0.0039940	0.0347811	-0.0018555	0.0005422
11	0.0008035	-0.0018555	0.0171455	-0.0005091
12	-0.0035791	0.0005422	-0.0005091	0.0064530

(b) OPDM of S_1

S9 (10e,12o) 6-roots SA-CASSCF energy (in Hartree) of involved π and π^* orbitals in $2\pi\pi^*$ state along MEP-PES

	π	π^*
FC	-0.3577	-0.0217
MEP Point 1	-0.3120	-0.0321
MEP Point 2	-0.3107	-0.0358
MEP Point 3	-0.3056	-0.0489
MEP Point 4	-0.2878	-0.0613
S_0/S_1 MECP	-0.2020	-0.0725

Table S1: π and π^* orbital energy (in Hartree) involved in $2\pi\pi^*$ state of Ds along MEP. Due to non-planarity in six-member ring at S_0/S_1 MECP, the energy of π orbital increases and that of π^* decreases gradually along the pathway.

S10 Effect of basis sets on VEEs calculated at TD-DFT/b3lyp level of theory at S_0 minima

States	6-31g	6-31+g(d)	6-311++g(d,p)	cc-pVTZ	Nature of excitation
S_1	4.09 (0.4844)	3.89 (0.0321)	3.88 (0.0005)	3.91 (0.0368)	$1\pi\pi^*$
S_2	4.30 (0.4703)	4.35 (0.0400)	4.33 (0.0004)	4.34 (0.0493)	$2\pi\pi^*$
S_3	4.57 (0.4629)	4.45 (0.0389)	4.42 (0.0004)	4.40 (0.0473)	$1n\pi^*$
S_4	4.91 (0.4456)	4.72 (0.0373)	4.68 (0.0003)	4.70 (0.0441)	$3\pi\pi^*$

Table S2: Effect of basis sets on VEEs calculated at TD-DFT/b3lyp level of theory with O.S. given in the parenthesis.
